# Selection and characterization of a DNA aptamer for Patulin and its application in a label-free fluorescence assay for fruit juices

**DOI:** 10.1186/s44280-025-00101-2

**Published:** 2025-12-29

**Authors:** Sihan Wang, Jiayi Liang, Haiyang Jiang, Jianzhong Shen, Zhanhui Wang, Juewen Liu

**Affiliations:** 1 Department of Chemistry, Waterloo Institute for Nanotechnology, University of Waterloo, Waterloo, ON N2L 3G1 Canada; 2 Department of Veterinary Pharmacology and Toxicology, National Key Laboratory of Veterinary Public Health Security, College of Veterinary Medicine, China Agricultural University, Beijing, 100193 China

**Keywords:** Patulin, Aptamers, SELEX, Fruit juice, Biosensors

## Abstract

Patulin (PTL, 154 Da) is a small molecule toxin produced as a mold metabolite. PTL is unstable in animals and is quickly converted to its metabolites. Thus, few successful antibodies were reported even after extensive efforts in hapten design. In this work, a library immobilization strategy was used to obtain DNA aptamers for PTL, where exposure of PTL to live animals or cells is no longer needed. After 20 rounds of selection, the most abundant aptamer named PTL-1 has a dissociation constant (*K*_d_) of 12.5 μM based on isothermal titration calorimetry and 18.4 μM based on thioflavin T (ThT) fluorescence spectroscopy. PTL-1 has excellent selectivity against nine other antibiotics. Compared to previously reported aptamers for PTL, PTL-1 is either shorter or has higher affinity. A label-free ThT fluorescence assay based on PTL-1 was developed, which has a detection limit of 0.2 μM (detection range 0.2–20 μM) for PTL in fruit juices. The PTL-1 aptamer shows resistance to matrix interference and can be applied to the rapid detection of PTL in a blended juice. This study is a nice demonstration of selecting aptamers for a target molecule difficult for the production of antibodies.

**Graphical Abstract:**

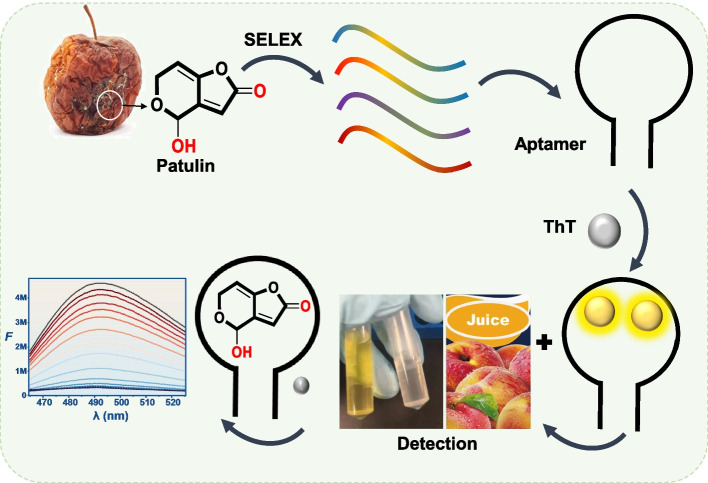

**Supplementary Information:**

The online version contains supplementary material available at 10.1186/s44280-025-00101-2.

## Introduction

Patulin (PTL) is a secondary metabolite produced by fungi such as *Penicillium expansum*, and *Aspergillus*. It is widely found in fruits and fruit products (e.g., juices, jams), vegetables, fodder, and grains, with apples and apple products being the most common [1]. PTL was first discovered in 1941 and was initially used as a broad-spectrum antibiotic. After intensive studies on its toxicity, PTL was found to damage the digestive system and central nervous system in humans. Therefore, PTL was reclassified as a mycotoxin in 1960 [2]. Consumption of PTL-contaminated food can cause serious harm to human health, especially in children. Many countries and regions have set limits for PTL contamination in food, with the U.S. Food and Drug Administration stipulating that the level of PTL in apple products be lower than 50 μg/kg [3]. In the EU and China, the limit of PTL in fruit juices and beverages is 50 μg/kg, and the limit is lowered to 10 μg/kg in apple-based baby food [4].

To achieve rapid detection of PTL, immunoassays have been attempted since 1986. However, due to difficulties associated with hapten synthesis and the poor in vivo stability of PTL, the resulting antibodies had low titers and poor recognition of free PTL [5]. Since the double bond in PTL is susceptible to reduction by sulfhydryl compounds (glutathione, etc.) in blood, it is difficult to retain intact PTL hapten structures in animals [6]. To date, few anti-PTL antibodies are available, and no PTL antibodies can meet the requirements for reliable detection of PTL.

Given the slow development of PTL antibodies, research on PTL aptamers might provide an alternative solution. Aptamers are single-stranded oligonucleotides that can selectively bind to target molecules [7–13]. A set of PTL aptamers based on a graphene oxide (GO) immobilization strategy was reported in 2016. Using a secondary enzyme for signal amplification, this aptamer system claimed a detection limit of 48 pg/mL PTL [14]. However, using GO for aptamer selection has a low efficiency [15]. Recently, another study used a hybridization method for library immobilization and obtained a 46-nucleotide (nt) PTL aptamer, which achieved a detection limit of 0.25 μM for PTL in fruit juice [16].

In this work, we first briefly reviewed efforts in hapten design to isolate antibodies for PTL. Then, an aptamer selection was performed [8, 17–20]. After characterization of the aptamers, a rapid quantification method for PTL in fruit juice was established using a label-free fluorescence detection strategy. This work highlights the advantage of aptamers for target molecules that are challenging for the production of antibodies, especially those suffer from stability problems under in vitro or in vivo conditions. At the same time, this work provides a high quality aptamer for PTL.

## Results and discussion

### Previous attempts to obtain antibodies for PTL

**Fig. 1:**
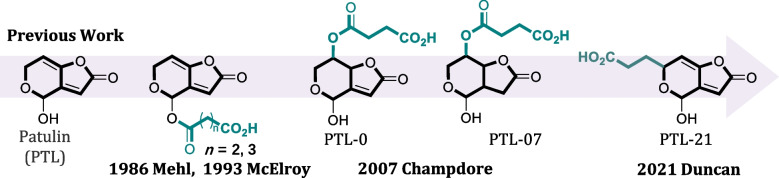
Some previously published PTL haptens for the production of antibodies

Extensive efforts have been made to raise antibodies for PTL, and some previous attempts to design haptens for PTL are shown in Fig. 1, where linkers have been added to various positions in the six-member ring structure. No attempts were made on the five-member ring since such modifications may significantly affect the binding of antibodies to unmodified PTL. The first reported attempts of PTL antibodies date back to 1986 [5], but the antibodies obtained had low titers and poor recognition of free PTL. To date, other researchers have designed a variety of haptens for PTL, but the performance of the antibodies obtained has been disappointing [2, 6, 21], and no monoclonal antibodies against PTL were obtained. There is still controversy as to whether anti-patulin antibodies produced are specific and suitable for use in rapid immunoassays (e.g., enzyme-linked immunosorbent assay and immunochromatographic test strips). A main challenge is that such haptens are susceptible to covalent modifications under in vivo conditions by reacting with thiol-containing molecules.

Thus, we turned our attention to aptamers, since the selection of aptamers does not need to involve live cells or animals. Aptamer selection can be achieved using either target immobilization or library immobilization methods. For target immobilization, targets similar to those shown in Fig. 1 can be covalently linked to a solid support [22]. Given the difficulties of using these haptens in antibody production, and covalent modification of PTL may block binding of aptamers from certain directions, we used the library immobilization strategy for aptamer selection [17, 18, 20, 23, 24].

### Selection of DNA aptamers for PTL

Selection of DNA aptamers for PTL was performed using a library containing a 30-nucleotide (nt) random region (Table S1). This library was hybridized with a biotinylated short single-stranded DNA, and the hybridized library was conjugated to streptavidin-modified agarose microspheres to form the capture-SELEX system (Fig. S1). The immobilized library was first subjected to buffer wash to remove non-immobilized sequences. PTL was then added to the immobilized library, and the eluted DNA was collected, which may contain aptamers that bind specifically to PTL [25]. For a total of 20 rounds of selection, the PTL concentration was 2.0 mM for rounds 1–16, 200 μM for round 17, and 20 μM for rounds 18–20. The progress of each round was monitored using real-time PCR (Fig. S2). We used the DNA collected from the last buffer wash and from adding PTL as templates for real-time PCR. A larger PCR cycle difference indicated more released DNA. Up to round 8 of the selection, their PCR cycle difference was within 1 cycle, indicating minimal aptamer enrichment. This cycle difference then progressive increased to 9 at round 16, indicating significant aptamer enrichment (approximately 2^9^-fold more DNA released by PTL than by buffer). Therefore, we dropped the PTL concentration from 2 mM to 200 µM at round 17, which still showed a 5-cycle difference. Further dropping the PTL concentration to 20 µM reduced the cycle to about 2 and this cycle difference did not improve by adding more selection cycles. Therefore, we stopped the selection at round 20. The round 20 library was sequenced, and the resulting sequences showed a high convergence, suggesting successful enrichment of aptamers.

**Fig. 2:**
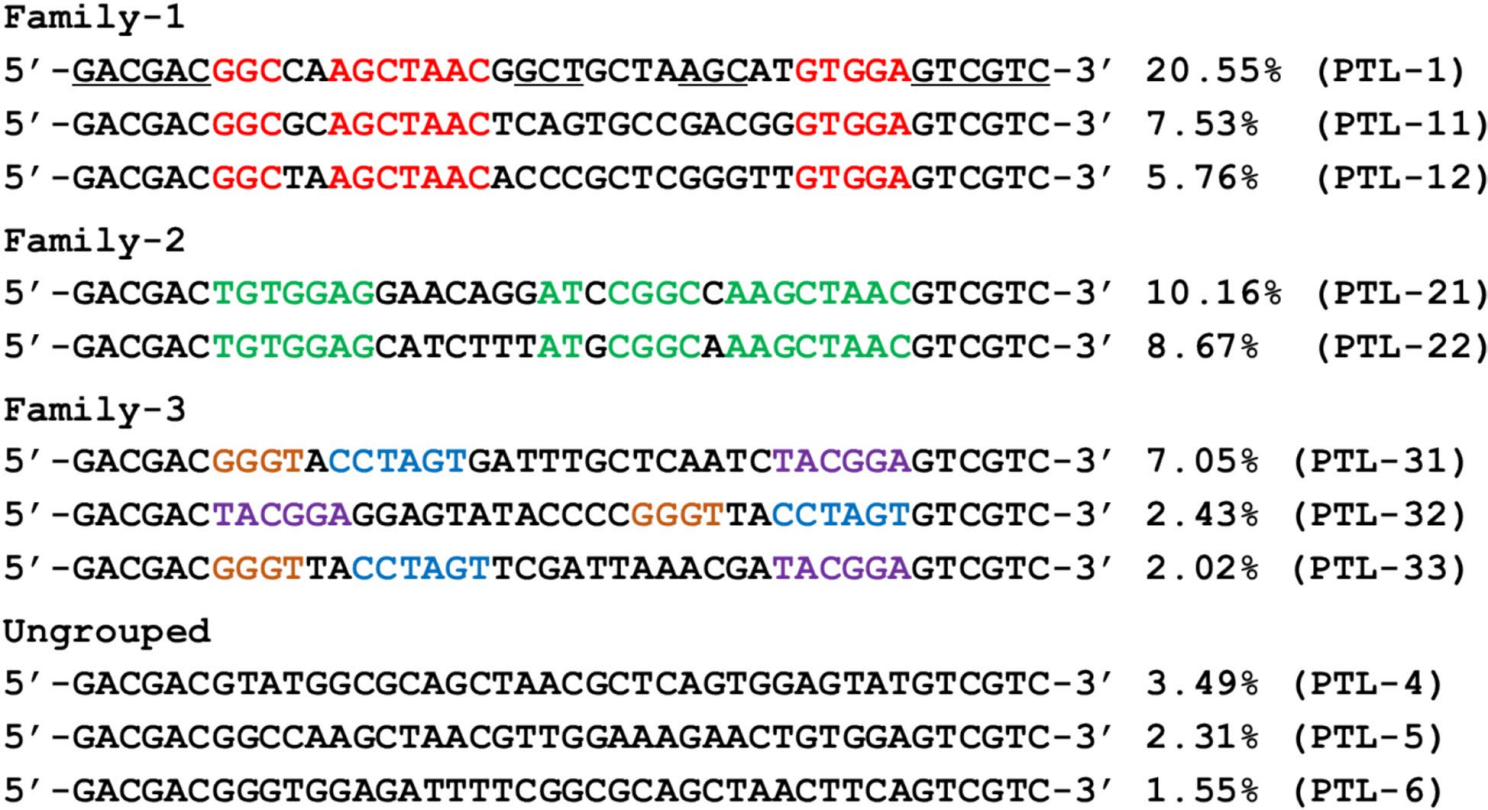
Family classification of PTL aptamers from the round 20 library. The base paired regions in PTL-1 are underlined

The top 11 enriched sequences were categorized into three families along with a few ungrouped sequences (Fig. 2). Among them, the most abundant PTL-1 DNA (belong to family 1) accounted for 20.55% of all enriched sequences. Nucleotides in red font are conserved regions. Family 2 also has very long conserved regions colored in green, whereas family 3 has a few shorter conserved regions. Based on the alignment of sequences, this is likely to be a successful selection.

### Sequence comparison with previously reported PTL aptamers

Before this work, two other labs have reported aptamers for PTL. The most recent work was from Cheng et al., [16] and they used the same capture-SELEX method. Their PTL concentration was gradually decreased from 200 µM to 10 µM. In addition, we used pH 6.0 for the selection, whereas they used pH 7.5. Interestingly, their most abundant family was similar to our family 3, where each sequence contained the same conserved regions (Fig. S3). In our case, the family 1 sequences are more abundant and thus we focused on this sequence, although we could not find our family 1 sequences in their published sequencing result.

The aptamers reported by Wu et al. [14] were obtained using the GO-SELEX method, and they reported 80-mer aptamer sequences that were not truncated. After a careful analysis, we did not find much relationship between their sequences and ours (Table S2). Given the length of their sequences and a lack of well-defined secondary structures, we did not compare with their sequences further.

### Binding affinity of aptamers to PTL

**Fig. 3:**
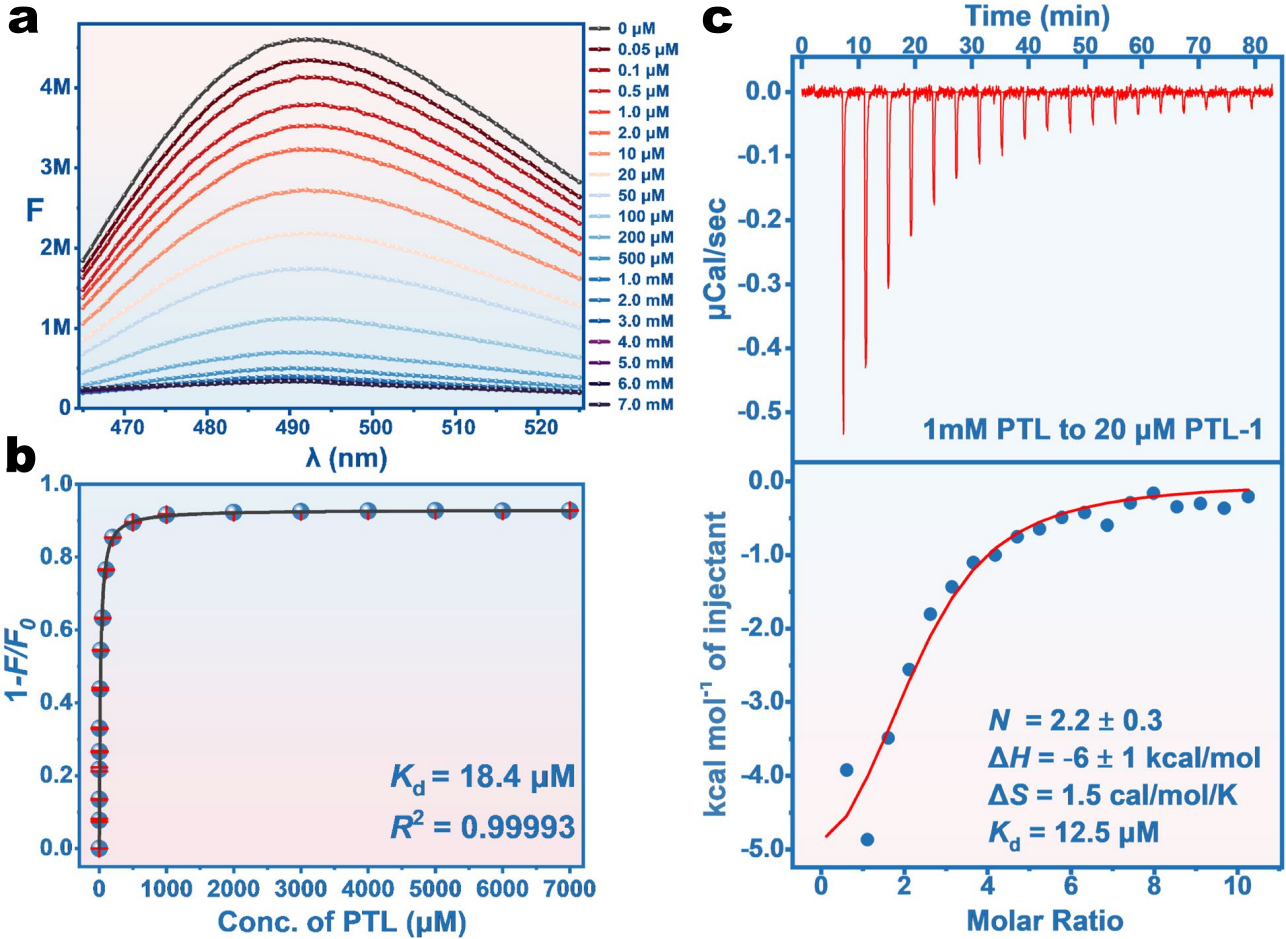
**a** Fluorescence emission spectra of 1.0 μM PTL-1 and 2.0 μM ThT upon titration of various concentrations of PTL. **b** Fitting of a binding curve based on the ThT fluorescence titration. **c** Measurement of binding of PTL by PTL-1 aptamer using ITC. *ThT* Thioﬂavin T, *ITC* Isothermal titration calorimetry

To evaluate aptamer binding to PTL, thioflavin T (ThT) fluorescence spectroscopy was used. ThT dye does not fluoresce on its own, but it can produce a fluorescence signal after non-specific binding to an aptamer [26]. After mixing PTL-1 with ThT, the initial fluorescence signal *F*_0_ was recorded. Upon the addition of PTL, a fraction of ThT might be displaced from the aptamer chain, causing a decrease in the fluorescence intensity (*F*). As shown in Fig. 3a, the fluorescence signal gradually decreased with increasing PTL concentration in the range of 0.05 μM–7.0 mM. The fluorescence signals corresponding to each PTL concentration were plotted (Fig. 3b) and fitted by Eq. 1 yielding an apparent *K*_d_ of 18.4 μM.

The affinity of PTL-1 was also determined by isothermal titration calorimetry (ITC), which is a highly reliable method for label-free binding assay [27]. Based on the *K*_*d*_ data measured by ThT, 20 μM PTL-1 was titrated using 1.0 mM PTL. As shown in Fig. 3c, titrating PTL into the PTL-1 aptamer produced exothermic peaks, with with *ΔH* = −6 kcal/mol, and *ΔS* = 1.5 cal/mol·K (−T*ΔS = *−0.45 kcal/mol). Therefore, the binding of the PTL-1 aptamer to PTL is driven by both enthalpy and entropy with contributions from enthalpy being dominant [28]. The measured *K*_d_ from ITC value was 12.5 μM, which was close to the 18.4 μM from the ThT fluorescence spectroscopy.

The Stojanovic group recently examined the effect of target structure and aptamer affinity [17], and it is clear from that work that there is an affinity limit for each target [8]. For example, the *K*_d _values for the glucose and acrylamide aptamers are about 5 mM, and for lactate 0.4 mM [29]. Metronidazole has a similar size to PTL and its aptamer has a *K*_d _of 17 μM from ITC [30]. PTL is a small molecule with few binding epitopes crowded in a small space. Thus, this 20 to 30 μM binding affinity is quite reasonable based on its structural features.

### Specificity of the PTL-1 aptamer

**Fig. 4:**
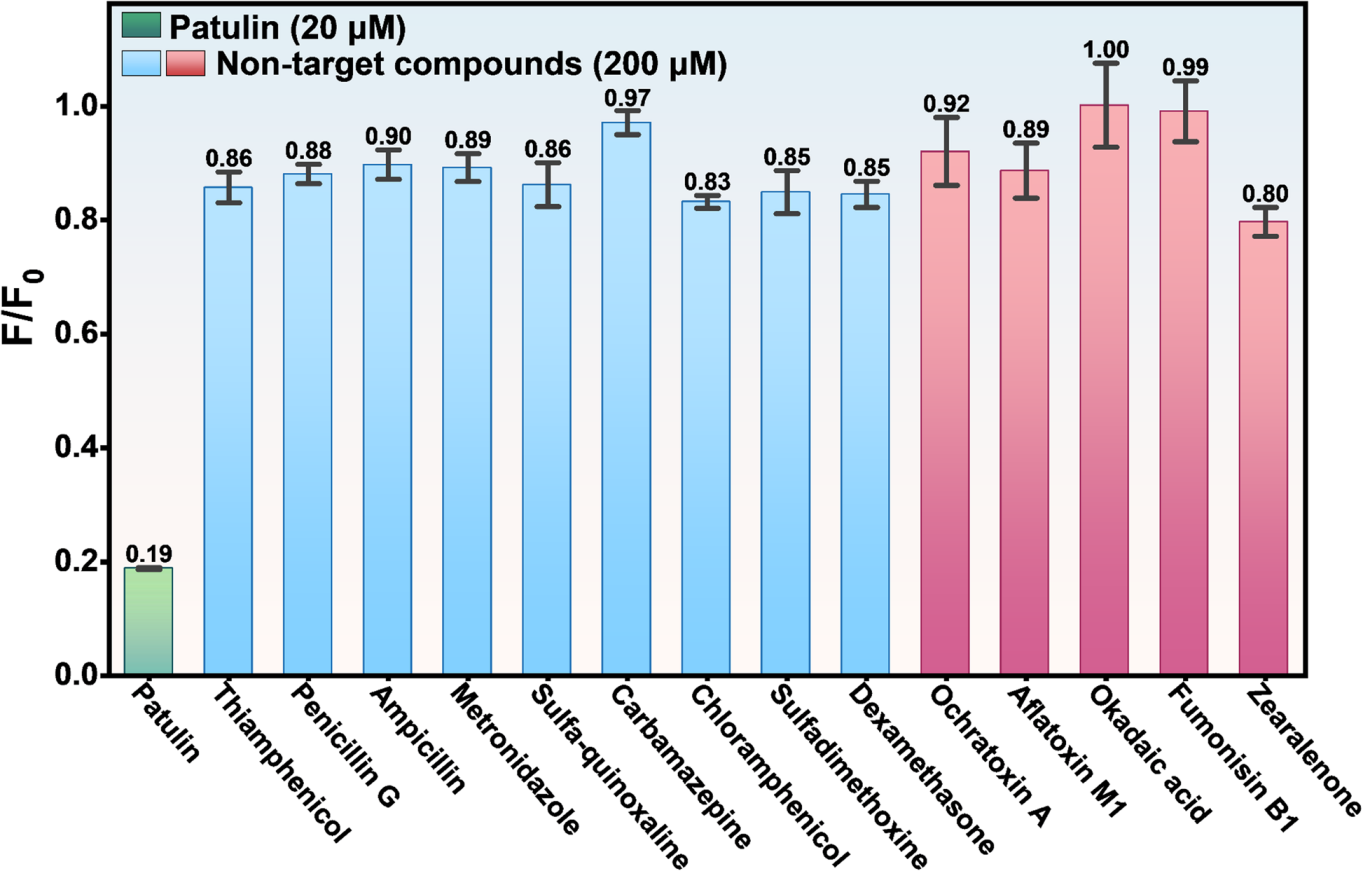
Selective testing of PTL-1 based on ThT fluorescence assay

To further investigate the specificity of the PTL-1 aptamers, 14 small molecules (thiamphenicol, penicillin-G, ampicillin sodium, metronidazole, sulfa-quinoxaline sodium, carbamazepine, chloramphenicol, sulfadimethoxine, dexamethasone, ochratoxin A, aflatoxin M1, okadaic acid, fumonisin B1, and zearalenone) were tested for their ability to quench the PTL-1 + ThT fluorescence system. These molecules were tested at 200 μM, which was tenfold higher compared to that of PTL (20 μM). The ThT fluorescence test in Fig. 4 revealed no significant decrease in fluorescence intensity with the addition of these nine compounds. In contrast, the fluorescence decreased significantly upon the addition of PTL. This result indicates that the PTL-1 aptamer has good selectivity for PTL.

### Aptamer mutation for secondary structure analysis

**Fig. 5:**
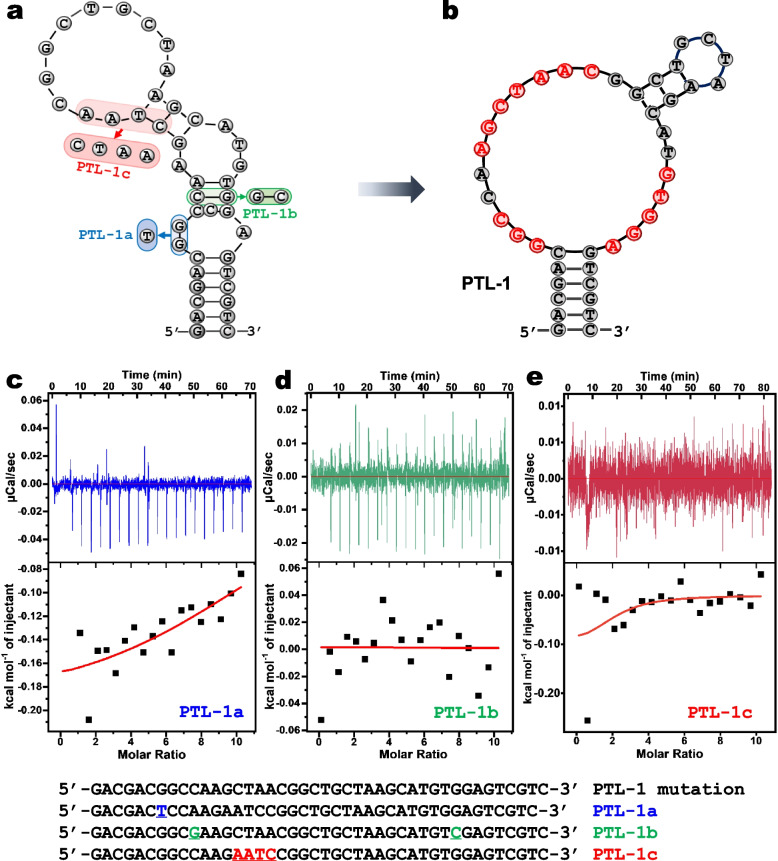
**a** *mFold* predicted secondary structure of PTL-1 and the three base mutation sites. **b** Secondary structure of PTL-1 redefined by conserved region mutation analysis. ITC results of mutants (**c**) PTL-1a; (**d**) PTL-1b; and (**e**) PTL-1c

Using *mFold*, the secondary structure of PTL-1 was predicted to be "L-shaped" with three loop regions (Fig. 5a). A lot of the conserved nucleotides are in base paired regions, and thus this predicted structure may not reflect the actual binding structure. To better understand its structures, we performed a few mutation studies. To explore the effect of the base-pairing of the aptamer stem on binding, we designed a mutant named PTL-1a, where the two guanine bases at positions 7 and 8 were mutated to a T base, which paired with the A base at position 35. The binding of PTL-1a was characterized by ITC (Fig. 5c) and no binding was observed, suggesting that this is position cannot be base paired. We then changed the G-C base pairs at positions 10 and 34 in the original structure to a C-G base pairs to make mutant 2 (PTL-1b), which was also failed to bind (Fig. 5d). Thus, this base pair should not be paired in the binding structure either. Finally, we mutated conserved regions by changing the CTAA to AATC to make mutant 3 (PTL-1c), which cannot bind to PTL as well (Fig. 5e). Therefore, we can infer that the three of the above conserved sequences are binding site locations for PTL and they should not be in base paired regions. Therefore, we believe that the structure shown in Fig. 5b is a more precise reflection of the secondary structure of the PTL-1 aptamer. This proposition is also supported by the sequence analysis for family 1, where a stem-loop structure can be formed in the non-conserved regions in all the sequences (Fig. 2).

### Effect of salt and pH on aptamer affinity

**Fig. 6:**
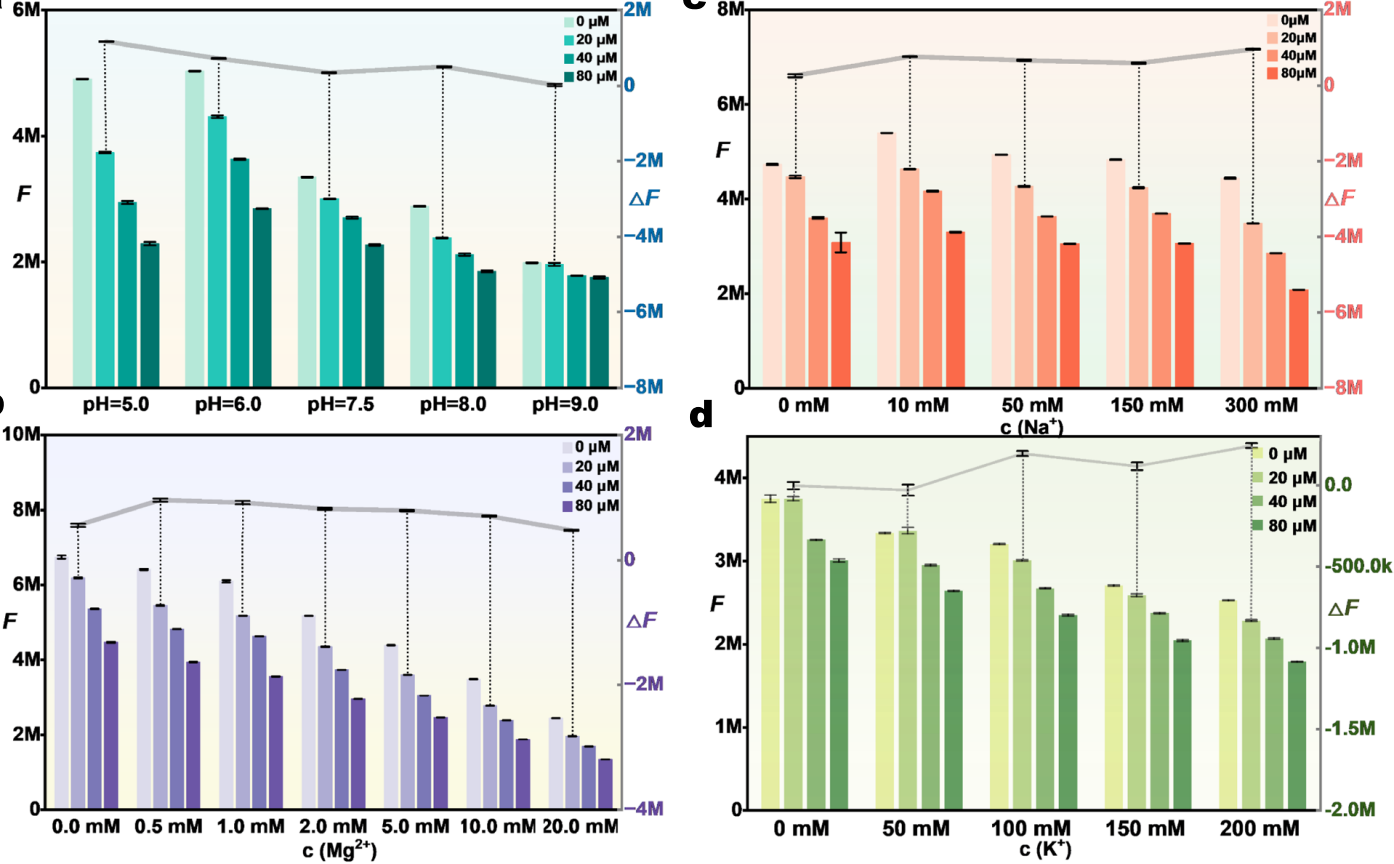
Titration of PTL to PTL-1 + ThT in different conditioned buffers (PTL concentrations: 0, 20, 40, 80 μM). **a** titration tests in different pH buffers; **b** titration tests with different Mg^2+^ concentrations in buffers (MgCl_2_ = 2.0 mM, KCl = 0 mM, pH = 6.0); **c** titration tests with different Na^+^ concentrations in buffers (NaCl = 150 mM, KCl = 0 mM, pH = 6.0); **d** titration tests with different K^+^ concentrations in buffers (NaCl = 150 mM, MgCl_2_ = 2.0 mM, pH = 6.0)

For detection, it is important to understand aptamer binding in different buffer environments. For this purpose, the fluorescence changes of PTL-1 + ThT were observed in different buffers to investigate the effects of pH and ionic strength. Under each condition, PTL was titrated at concentrations of 0, 20, 40, and 80 μM, and the results are shown in Fig. 6. The results indicate the *F*, which gradually decreased with increasing PTL concentration. The gray line indicates the fluorescence difference (*ΔF*) between 0 μM and 20 μM. At the pH of the solution system ranging from 5.0 to 9.0 (Fig. 6a), the initial fluorescence signal was attenuated as the solution pH increased. The results showed that PTL-1 was more stable in binding to PTL in neutral and acidic environments and was almost inactivated in the alkaline condition (pH = 9.0).

The effect of metal ions (Na^+^, Mg^2+^, and K^+^) is shown in Fig. 6b–d. The increase of Na^+^ concentration has little effect on the affinity of PTL-1, and a high Na^+^ concentration resulted in slightly more fluorescence signal change (Fig. 6b). However, high concentrations of Mg^2+^ weakened the initial fluorescence signal (Fig. 6c). We hypothesized that Mg^2+^ screened charge repulsion and weakened the binding between ThT and negatively charged aptamers, and Mg^2+^ may even serve as a fluorescence quencher. Under physiological condition of 1–2 mM Mg^2+^, the sensor has an optimal performance. The increase of K^+^ concentration had a similar effect to that of Na^+^, although the initial fluorescence appeared to be more quenched (Fig. 6d). The above titration results indicate that the PTL-1 aptamer can maintain a stable affinity in multiple buffer systems, especially in weakly acidic environments.

### Comparison of aptamer performance

A previous paper studied an aptamer similar to our family 3 sequence, and we studied one of our family 1 sequence. To have a side-by-side comparison, we also measured the binding of previously reported PAT-6 (similar to our family 3 sequences). Using both ITC and ThT fluorescence tests, at pH 6.0, PAT-6 has weaker binding affinities (*K*_*d*_ = 48 μM by ThT, Fig. 4S). Thus, our family 1 aptamer PTL-1 has overall higher affinity. Both selections gradually decreased the PTL concentration, and pH might be responsible for the enrichment of our family 1 sequences.

### Detection in juice samples

With a PTL aptamer in hand, many methods are available to convert it to biosensors [31], and we are particularly interested in optical sensors for its simplicity [9, 32–34]. In this work, a proof-of-concept detection using ThT fluorescence was tested. Since fruit products are the main samples for the detection of PTL, commercially available juices were first diluted tenfold for assays. PTL-1 (1.0 μM) and ThT dye were added directly. The principle of blind sample addition was adopted and different concentrations of PTL standards were added to the sample matrix.

**Fig. 7:**
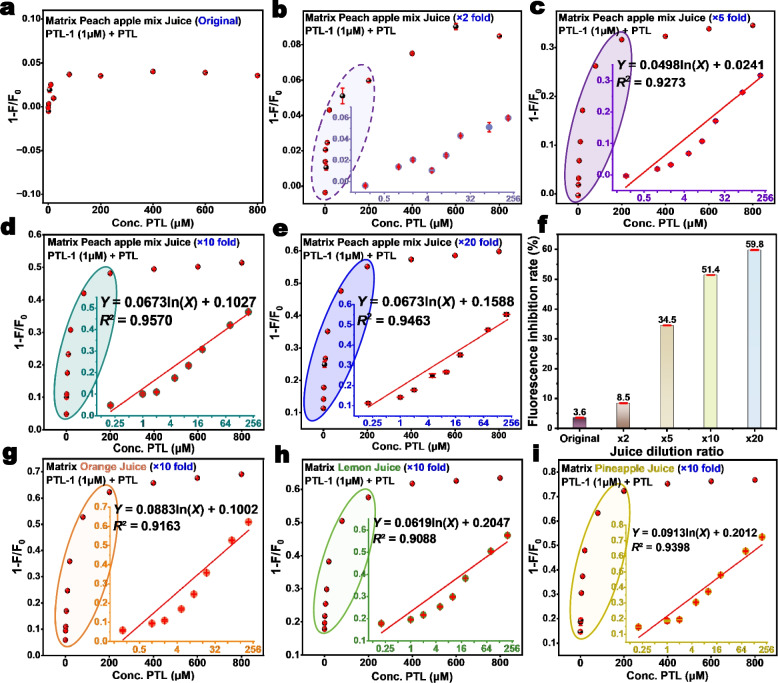
Rapid detection of PTL in fruit juices by PTL-1 + ThT fluorescent probe. Detection in peach apple mixed juice (**a**) without any dilution; **b** with twofold dilution; **c** with fivefold dilution; **d** with tenfold dilution; and **e** with 20-fold dilution. **f** Patulin-induced PTL-1 and ThT fluorescence drop rate in fruit juices at different dilution ratios; Detection in tenfold diluted (**g**) orange juice; **h** lemon juice; and **i** pineapple juice

The sensitivity of PTL detection varied across different dilution ratios and types of fruit juice. When the PTL-1 aptamer was used for ThT fluorescence detection in undiluted fruit juice, the fluorescence dropped only approximately 5% (Fig. 7a). Since direct detection using this method had limited sensitivity, we then tried dilution. The detection results after a two-fold dilution, the fluorescence change slightly improved (Fig. 7b). When the juice was diluted five-fold, the detected fluorescence drop exhibited a better linear relationship with the concentration of PTL, with a detection range of 0.2–200 μM and the limit of quantification (LOQ) = 0.2 μM (based on S/N = 10). However, the fluorescence decrease rate at this point is only 30% (Fig. 7c). When diluted tenfold (Fig. 7d), the linear detection range for PTL is 0.2–200 μM, with a LOQ of 0.2 μM. When the juice dilution ratio is tenfold and 20-fold, the fluorescence quenching efficiency of PTL-1 + ThT exceeds 50% in both cases (Fig. 7e, f). Samples diluted 5-, 10-, and 20-fold exhibited pH values ranging from 5.4 to 5.8, which are closer to the selection buffer. Consequently, these samples exhibited excellent detection linearity and sensitivity. Since dilution of the juice also diluted the PTL target, considering operational feasibility, a tenfold dilution was determined to be an optimal choice. Under this condition, the PTL-1 aptamer achieved a LOQ of 0.2 μM for PTL in orange juice (Fig. 7g), lemon juice (Fig. 7h), and pineapple juice (Fig. 7i), with a detection range of 0.2–200 μM.

The main goal of this work is to obtain a reliable aptamer for PTL and therefore, and we did not try to use advanced signal amplification methods to improve sensitivity. For example, using the adenosine triphosphate aptamer, which has a similar *K*_d_ of approximately 10 μM, many biosensors have been reported to have nM or even lower limit of detection (LOD). When coupled with various nanomaterials and catalytic amplification, this PTL aptamer should also achieve better sensitivity.

## Conclusions

As a highly toxic contaminant in fruits and vegetables, PTL has long lacked a rapid and sensitive detection method, and its detection still relies mainly on laboratory instrumentation methods. In this study, we isolated DNA aptamers (PTL-1) with good affinity (*K*_*d*_ = 12.5 μM) for PTL in a slightly acidic matrix by using the capture-SELEX strategy. Simply using ThT as a fluorescent probe, the PTL-1 aptamer achieved rapid quantification of PTL in fruit juices with a LOD of 0.2 μM (tenfold diluted juices). Comparisons with previously reported PTL aptamers have also been made. This study provides a useful reference for the screening of aptamers for small molecule targets that are difficult to raise antibodies. This aptamer will likely find useful analytical applications for the detection of PTL, especially when coupled with advanced signal amplification strategies.

## Materials and methods

### Chemicals

All DNA sequences (Table S1) were purchased from Integrated DNA Technologies (Coralville, IA, USA). PTL (CAS: 149–29-1) was purchased from Aladdin Inc., Shanghai, China. 2-(4-Morpholino) ethanesulfonic (MES) sodium salt and free acid, sodium chloride, and magnesium chloride were purchased from Mandel Scientific (Guelph, ON, Canada). All the solutions were prepared using Milli-*Q* water. Juice samples were purchased from a local supermarket.

### Capture-SELEX

The library-immobilized aptamer selection strategy is based on previous publications [35–37]. Target was prepared in selection buffer (10 mM MES, pH 6.0, 150 mM NaCl, 5 mM MgCl_2_) at concentrations of 2 mM, 200 μM, and 20 μM, respectively. These solutions were stored at −20 °C for use. The DNA library was immobilized via hybridization to a capture strand attached to agarose beads. Before adding a target solution, the library-immobilized agarose beads were washed 12 times using the selection buffer to remove unbound or weakly adsorbed library strands. The PTL concentration was 2 mM for rounds 1–14, 200 μM for round 17, and 20 μM for rounds 18–20. The last round of eluted DNA collected was PCR amplified and purified for sequencing (McMaster University, Hamilton, ON, USA). DNA sequencing results were analyzed using Geneious Prime software (Version 2024.0.5, Auckland, New Zealand).

### Thioflavin T (ThT) fluorescence spectroscopy

ThT fluorescence spectroscopy was performed on a Horiba Fluoromax-4 fluorescence spectrophotometer (parameter:* E*_*x*_ = 420 nm, *E*_*m*_ = 490 nm). To 485 μL selection buffer, 5 μL aptamer (100 μM) and 10 μL ThT (100 μM) were added. Fluorescence measurements were performed in a quartz fluorescence cuvette (Ex: 420 nm; Em: 490 nm), and the initial fluorescence intensity was designated as *F*_*0*_. PTL was then titrated (1.0 μL each time) and the fluorescence *F* was measured at each PTL concentration (*c*) until the fluorescence intensity reached a steady value. The recorded data were analyzed and the apparent dissociation constant (*K*_d_) value was fitted using the following equation:

1 $$1-\frac F{F_0}=\frac{a\cdot c}{K_d+c}$$

### Isothermal titration calorimetry (ITC)

A MicroCal ITC200 instrument (Worcestershire, UK) was used for ITC. In a typical experiment, 300 μL target (1.0 mM of PTL dissolved in selection buffer) and aptamer (20 μM, annealed at 95 °C for 3 min followed by cooling to room temperature) were respectively prepared. Then, the target solution was drawn into a syringe, and the aptamer was loaded into the sample cell for a total 20 titrations (2.0 μL per drop, and 200 s intervals at 25 ℃). Concentration-heat curves obtained by titration were fitted and analyzed using the *MicroCal* 2.0 software accompanying the ITC instrument.

### Juice sample testing

Based on the ThT fluorescence method, juices were purchased from a local supermarket. The juices were diluted tenfold with water, and different concentrations of PTL standards (0.1–600 μM) were added. The fluorescence intensity of each sample was measured as a function of PTL concentration, and a standard curve was plotted to determine the detection limit.

## Supplementary Information

Supplementary Material 1. Fig. S1. Scheme of screening PTL aptamers based on the capture-SELEX methodology. Fig. S2. Real-time PCR cycle differences for 1–20 rounds of PTL aptamer selection for DNA eluted by buffer and by PTL. A lower PCR cycle number of PTL elution compared to buffer elution indicates better aptamer enrichment. Fig. S3. The secondary structure of the patulin aptamer (PAT-6) reported by Cheng et al. This sequence is similar to our family 3 aptamers. Fig. S4. Binding assays for a previously published aptamer. a ThT fluorescence results using 1.0 µM PAT-6 aptamer in 20 mM MES buffer, pH = 6.0, with 150 mM NaCl and 2.0 mM MgCl_2_; b ITC titration results. Note that due to its high *K*_d_ value and the low aptamer concentration used, this *K*_d_ determined using ITC is not accurate. Nevertheless, the data showed that the *K*_d_ of PAT-6 is higher than the PTL-1 aptamer. Table S1. The DNA sequences used in this study for aptamer selection. Table S2. Patulin family sequences from a GO-SELEX experiment reported by Wu et al. Only the middle 40 nucleotide random regions are shown, and the full aptamer need to add the two primer binding regions with a total of approximately 80 nucleotides.

## Data Availability

The data that support the findings of this study are available from the Federated Research Data Repository, at 10.20383/103.01367.

## Abbreviations

PTL: Patulin
ThT: Thioflavin T
SELEX: Systematic evolution of ligands by exponential enrichment
GO: Graphene oxide
ITC: Isothermal titration calorimetry
ELISA: Enzyme linked immunosorbent assay
PCR: Polymerase chain reaction
LOD: Limit of detection
LOQ: Limit of quantitation
S/N: Signal-to-noise ratio
*N * (in ITC): Stoichiometric number
*ΔH*: Enthalpy change
*ΔS*: Entropy change
*K*d: Affinity dissociation constant
