# Supplementary material for: Selection and characterization of a DNA aptamer for Patulin and its application in a label-free fluorescence assay for fruit juices
Source: One Health Adv. 2025 Dec 29;3(1):37. doi: 10.1186/s44280-025-00101-2 (PMC12745320; doi:10.1186/s44280-025-00101-2)
Supplement: Supplementary file 1 — Supplementary Material 1. Fig. S1. Scheme of screening PTL aptamers based on the capture-SELEX methodology. Fig. S2. Real-time PCR cycle differences for 1–20 rounds of PTL aptamer selection for DNA eluted by buffer and by PTL. A lower PCR cycle number of PTL elution compared to buffer elution indicates better aptamer enrichment. Fig. S3. The secondary structure of the patulin aptamer (PAT-6) reported by Cheng et al. This sequence is similar to our family 3 aptamers. Fig. S4. Binding assays for a previously published aptamer. a ThT fluorescence results using 1.0 µM PAT-6 aptamer in 20 mM MES buffer, pH = 6.0, with 150 mM NaCl and 2.0 mM MgCl2; b ITC titration results. Note that due to its high Kd value and the low aptamer concentration used, this Kd determined using ITC is not accurate. Nevertheless, the data showed that the Kd of PAT-6 is higher than the PTL-1 aptamer. Table S1. The DNA sequences used in this study for aptamer selection. Table S2. Patulin family sequences from a GO-SELEX experiment reported by Wu et al. Only the middle 40 nucleotide random regions are shown, and the full aptamer need to add the two primer binding regions with a total of approximately 80 nucleotides. [file 44280_2025_101_MOESM1_ESM.docx]

**Supporting Information:**

**Selection and Characterization of a DNA Aptamer for Patulin and Its Application in a Label-Free Fluorescence Assay for Fruit Juices**

Sihan Wang^1^, Jiayi Liang^1^, Haiyang Jiang^2^, Jianzhong Shen^2^, Zhanhui Wang^2*^ and Juewen Liu^1*^

^1^ Department of Chemistry, Waterloo Institute for Nanotechnology, University of Waterloo, Waterloo, Ontario N2L 3G1, Canada

^2^ Department of Veterinary Pharmacology and Toxicology, National Key Laboratory of Veterinary Public Health Security, College of Veterinary Medicine, China Agricultural University, Beijing 100193, China

**Buffer solution conditions**

Selection buffer: 20 mM MES, 150 mM NaCl, 2.0 mM MgCl_2_, pH = 6.0

Separation buffer: 20 mM MES, 150 mM NaCl, 2.0 mM, pH = 6.0


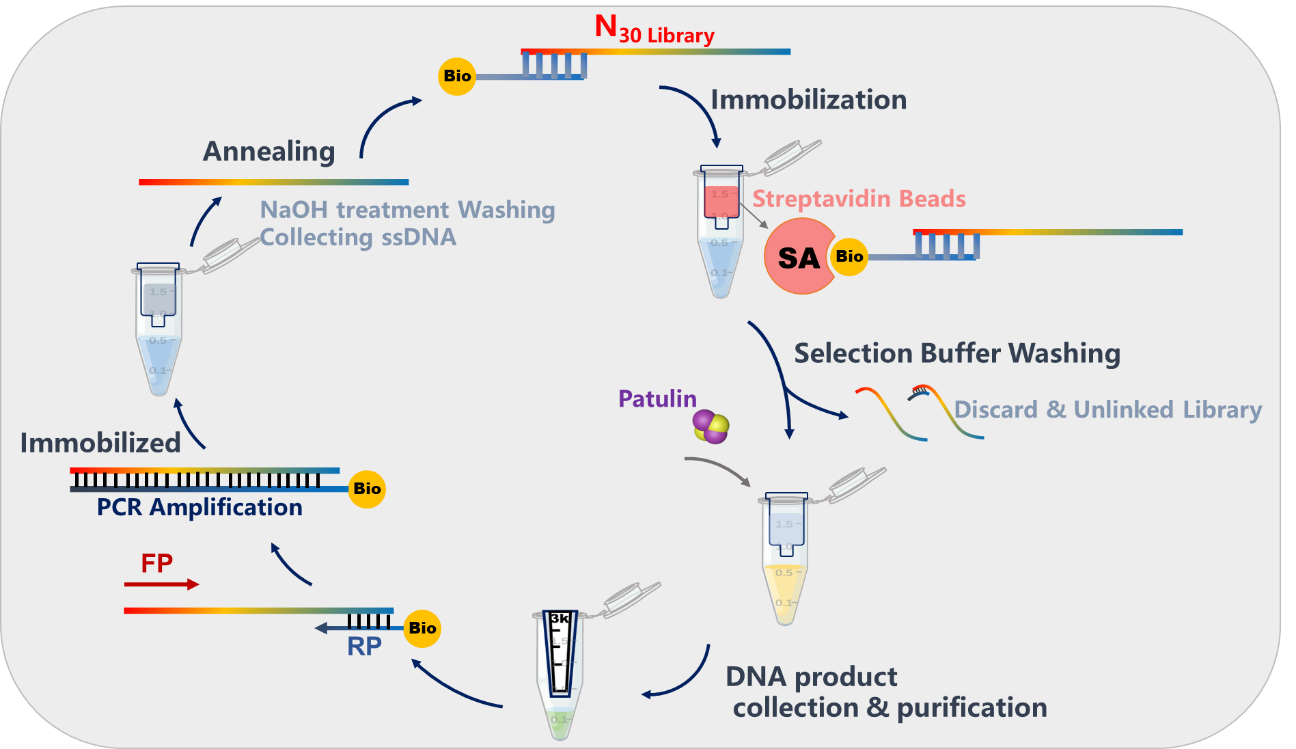


**Fig. S1**. Scheme of screening PTL aptamers based on the capture-SELEX methodology


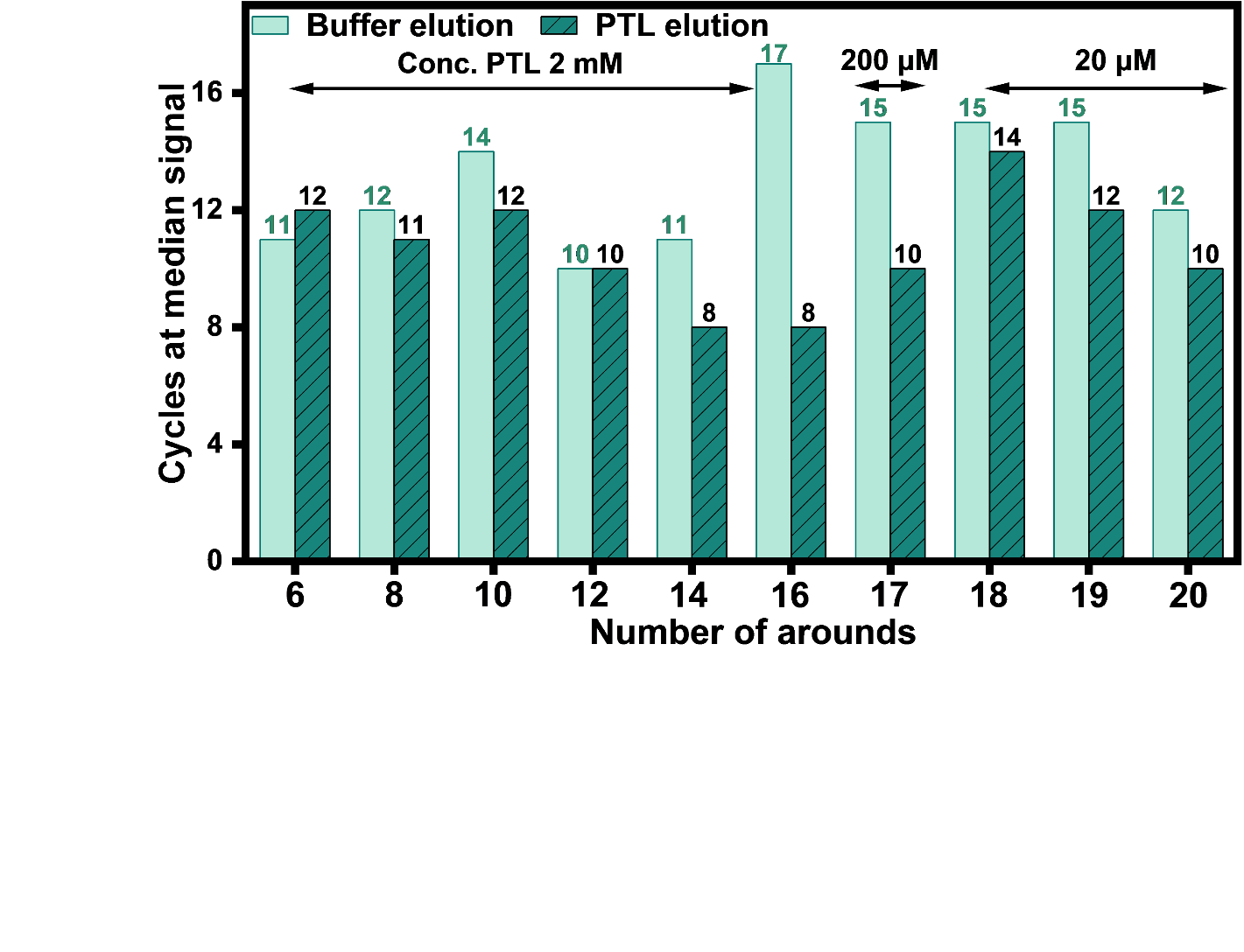


**Fig. S2.** Real-time PCR cycle differences for 1–20 rounds of PTL aptamer selection for DNA eluted by buffer and by PTL. A lower PCR cycle number of PTL elution compared to buffer elution indicates better aptamer enrichment


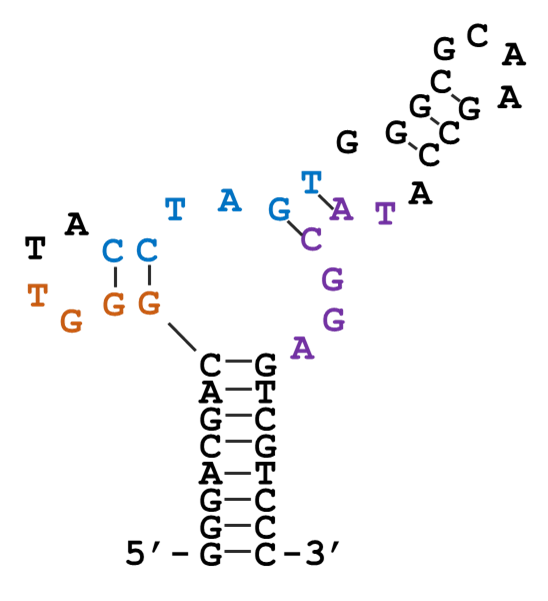


**Fig. S3.** The secondary structure of the patulin aptamer (PAT-6) reported by Cheng et al.^1^ This sequence is similar to our family 3 aptamers


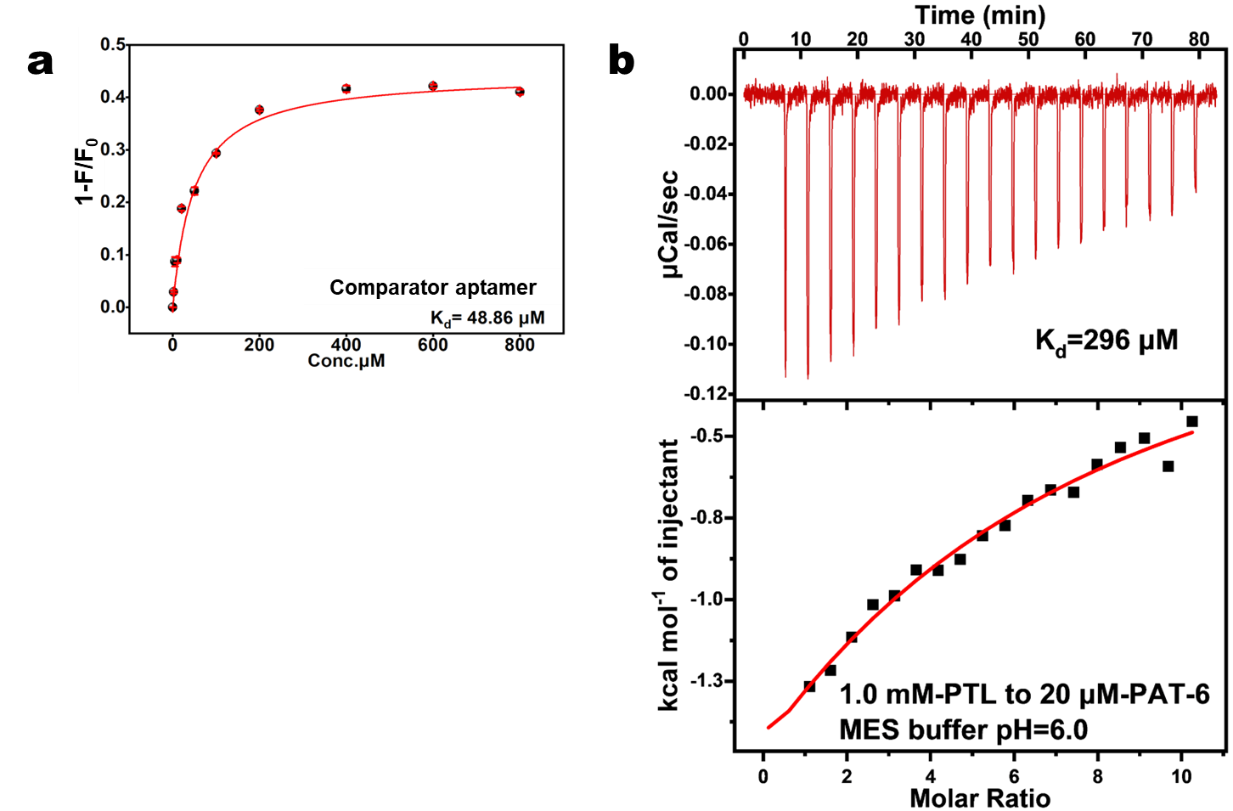


**Fig. S4.** Binding assays for a previously published aptamer. **a** ThT fluorescence results using 1.0 µM PAT-6 aptamer in 20 mM MES buffer, pH = 6.0, with 150 mM NaCl and 2.0 mM MgCl_2_; **b** ITC titration results. Note that due to its high *K*_d_ value and the low aptamer concentration used, this *K*_d_ determined using ITC is not accurate. Nevertheless, the data showed that the *K*_d_ of PAT-6 is higher than the PTL-1 aptamer

**Table S1.** The DNA sequences used in this study for aptamer selection

| **DNA** | **Sequence** |
| --- | --- |
| **Library** | **5′-GGAGGCTCTCGGGACGAC_(18)_-N_30_-GTCGTCCCGCCTTTAGGATTTACAG_(25)_-3′**  **Molar extinction coefficient: 690275 L/mol.cm** |
| **Fwd** | **5′-GGAGGCTCTCGGGACGAC-3′** |
| **Rev** | **5′-(bio)CTGTAAATCCTAAAGGCGGGACG-3′** |
| **Patulin**  **aptamers** | **Family-1**  **5’-GACGACGGCCAAGCTAACGGCTGCTAAGCATGTGGAGTCGTC-3’ 20.55% (PTL-1)**  **5’-GACGACGGCGCAGCTAACTCAGTGCCGACGGGTGGAGTCGTC-3’ 7.53% (PTL-11)**  **5’-GACGACGGCTAAGCTAACACCCGCTCGGGTTGTGGAGTCGTC-3’ 5.76% (PTL-12)**  **Family-2**  **5’-GACGACTGTGGAGGAACAGGATCCGGCCAAGCTAACGTCGTC-3’ 10.16% (PTL-21)**  **5’-GACGACTGTGGAGCATCTTTATGCGGCAAAGCTAACGTCGTC-3’ 8.67% (PTL-22)**  **Family-3**  **5’-GACGACGGGTACCTAGTGATTTGCTCAATCTACGGAGTCGTC-3’ 7.05% (PTL-31)**  **5’-GACGACTACGGAGGAGTATACCCCGGGTTACCTAGTGTCGTC-3’ 2.43% (PTL-32)**  **5’-GACGACGGGTTACCTAGTTCGATTAAACGATACGGAGTCGTC-3’ 2.02% (PTL-33)**  **Ungrouped**  **5’-GACGACGTATGGCGCAGCTAACGCTCAGTGGAGTATGTCGTC-3’ 3.49% (PTL-4)**  **5’-GACGACGGCCAAGCTAACGTTGGAAAGAACTGTGGAGTCGTC-3’ 2.31% (PTL-5)**  **5’-GACGACGGGTGGAGATTTTCGGCGCAGCTAACTTCAGTCGTC-3’ 1.55% (PTL-6)** |
| **mutants** | **5’-GACGACTCCAAGAATCCGGCTGCTAAGCATGTGGAGTCGTC-3’ (PTL-1a)**  **5’-GACGACGGCGAAGCTAACGGCTGCTAAGCATGTCGAGTCGTC-3’ (PTL-1b)**  **5’-GACGACGGCCAAGAATCCGGCTGCTAAGCATGTGGAGTCGTC-3’ (PTL-1c)** |

**Table S2.** Patulin family sequences from a GO-SELEX experiment reported by Wu et al.^2^ Only the middle 40 nucleotide random regions are shown, and the full aptamer need to add the two primer binding regions with a total of approximately 80 nucleotides.

| Aptamer | Sequence | *K*_d_ (nM) |
| --- | --- | --- |
| PAT-4 | **TCCCAACATTCGCACTTTGTATTCCCATTCGTTTAGCCCT** | 59 |
| PAT-6 | **GGCCCCGATATGATTGCTATCATTCGCATCTGCTTCACTC** | 68 |
| PAT-11 | **GGCCCGCCAACCCGCATCATCTACACTGATATTTTACCTT** | 21 |

**References**

1. Cheng J, Liu X, Liu S, Liu H, Wu Q, Hou J, et al. Selection and characterization of novel aptamers specific for patulin and label-free split sensing. Microchem J. 2024;207:112118. https://doi.org/10.1016/j.microc.2024.112118.

2. Wu S, Duan N, Zhang W, Zhao S, Wang Z. Screening and development of DNA aptamers as capture probes for colorimetric detection of patulin. Anal Biochem. 2016;508:58–64. https://doi.org/10.1016/j.ab.2016.05.024.
